# Dissociation in SLE: A part of lupus fog?

**DOI:** 10.1177/09612033211050347

**Published:** 2021-10-29

**Authors:** Rory C Monahan, Anne ME Blonk, Esther Baptist, Huub AM Middelkoop, Margreet Kloppenburg, Tom WJ Huizinga, Nic J van der Wee, Gerda M Steup-Beekman

**Affiliations:** 1 Department of Rheumatology, 4501 Leiden University Medical Center, the Netherlands; 2 Department of Psychiatry, Leiden University Medical Center, the Netherlands; 3 Department of Psychiatry, Haaglanden Medical Center, the Hague, the Netherlands; 4 Department of Neurology 4501 Leiden University Medical Center, the Netherlands; 5 Department of Psychology, Health, Medical and Neuropsychology Unit, Leiden University, the Netherlands; 6 Department of Clinical Epidemiology, 4501 Leiden University Medical Center, the Netherlands; 7 Department of Rheumatology, Haaglanden Medical Center, the Hague, the Netherlands

## Abstract

**Introduction:**

Lupus fog is ill-defined. We aimed to study whether lupus fog is the result of dissociation by studying the prevalence of dissociation and dissociative fog in patients with SLE and neuropsychiatric manifestations of inflammatory and non-inflammatory origin.

**Methods:**

Patients visiting the tertiary referral center for neuropsychiatric systemic lupus erythematosus (NPSLE) of the LUMC between 2007–2019 were included. Patients were classified as having neuropsychiatric symptoms of inflammatory or non-inflammatory origin. Dissociation was studied using the Dissociative Experience Scale-II (DES), in which the presence of 28 dissociative symptoms is rated (0–100% of the time), of which one question assesses the presence of a dissociative fog directly. Average scores are calculated and scores ≥ 25 are considered indicative of a dissociative disorder. A score of ≥ 30 on question 28 (dissociative fog) was considered indicative for the presence of a fog. Summary scores in the general adult population range from 4.4 to 14. Multiple regression analysis (MRA) was performed to study the association between inflammatory neuropsychiatric symptoms and dissociation. DES results are presented as median (range) and MRA as B and 95% confidence interval (CI).

**Results:**

DES questionnaires were available for 337 patients, of which 69 had an inflammatory NPSLE phenotype (20%). Mean age in the total study population was 43 ± 14 years and the majority was female (87%). The median dissociation score was 7.1 (0–75) and did not differ between patients with neuropsychiatric symptoms of inflammatory or non-inflammatory origin (B: −0.04 (95% CI: −0.17; 0.09)). 35 patients (10%) had a score indicative of a dissociative disorder. The most common type of dissociation was absorption/imagination. 43 patients (13%) reported a dissociative fog.

**Discussion:**

In most patients with SLE and neuropsychiatric symptoms, dissociative symptoms are within normal range, regardless of underlying etiology. Dissociative fog is present, but uncommon. Lupus fog is most likely not associated with dissociation.

## Introduction

The term ‘lupus fog’ is used by many people with systemic lupus erythematosus (SLE). On patient fora and websites, confusion, difficulty planning, loss of concentration, difficulty in articulating thoughts, and memory impairment are symptoms described in the context of this fog. Despite the frequent occurrence of these symptoms, lupus fog has never been formally studied and there is no clear definition. Only two studies up to date mention lupus fog and describe it as periods of forgetfulness and confusion that is related to impaired cognition.^1,2^ Based on the type of complaints reported by patients in clinical practice and on patients’ websites and fora, we hypothesized that the symptoms mentioned as part of lupus fog might also be related to dissociation.

Dissociation is defined as a disruption, interruption, and/or discontinuity of the normal, subjective integration of one or more aspects of psychological functioning.^3^ The presence of dissociation can be evaluated by the Dissociative Experiences Scale (DES).^4^ One question of this scale assesses the presence of a fog directly: “Some people sometimes feel as if they are looking at the world through a fog, so that people and objects appear far away or unclear.” There are different mechanisms that might lead to dissociative symptoms, such as a (dissociative) fog, in patients with SLE. Although any person may experience dissociation to some degree, more severe dissociation is thought to be caused by (chronic) stress and/or trauma. As stress is common in patients with SLE, a higher level of dissociative symptoms may be present.^5,6^ It has even been suggested that posttraumatic stress disorder increases the risk of autoimmune diseases, including SLE.^7–9^ In addition, it has been shown that inflammation is associated with dissociation, possibly through the alterations of the hypothalamic–pituitary–adrenal axis.^10^ Lastly, psychiatric disorders and fatigue are common in patients with SLE, which are known to increase dissociative symptoms.^11^ These potential mechanisms might cause symptoms such as confusion and forgetfulness, which are described both in dissociation and lupus fog. Recognizing dissociative symptoms is of importance, as they are associated with a greater disease burden and reduced treatment outcomes.^12^

In this study, we aimed to explore our hypothesis that dissociation could be a component of lupus fog by studying the prevalence of dissociative symptoms (including dissociative fog) in patients with SLE. In addition, we aimed to assess the role of inflammation on dissociation by comparing dissociative symptoms in patients with SLE and neuropsychiatric symptoms of inflammatory and non-inflammatory origin.

## Methods

### Participants

Patients visiting the tertiary referral center for neuropsychiatric systemic lupus erythematosus (NPSLE) of the Leiden University Medical Center between 2007–2019 with informed consent and a clinical diagnosis of SLE were included. In the NPSLE clinic, patients are evaluated in a multidisciplinary setting over the course of one day. This multidisciplinary evaluation process has been described in detail previously.^13^ In short, patients are evaluated by the following specialisms: rheumatology, neurology, clinical neuropsychology, psychiatry, and vascular internal medicine. Other investigations include MRI assessment and extensive laboratory assessment. In a multidisciplinary meeting, consensus is reached regarding the underlying cause of the neuropsychiatric symptoms. Symptoms are attributed to SLE requiring treatment with immunosuppressive or anticoagulants (NPSLE) or to other causes and/or neuropsychiatric symptoms for which symptomatic treatment suffices (minor/non-NPSLE). If NPSLE diagnosis is established, the 1999 American College of Rheumatology NPSLE case definitions are assigned. In addition, NPSLE phenotype is assigned based on the suspected underlying pathogenetic mechanism (inflammatory, ischemic, and combined), for which clinical, radiological, and laboratory features are taken into account. Patients in whom no consensus was reached were excluded. For this study, patients were categorized as having neuropsychiatric symptoms of inflammatory origin (inflammatory or combined phenotype) or non-inflammatory origin (ischemic NPSLE and minor/non-NPSLE; non-inflammatory phenotype). This study was approved by the local medical ethical committee.

### Data collection

Clinical information, including patient demographics, diagnosis of SLE and NPSLE, and medication use, was obtained during multidisciplinary assessment. Disease activity was calculated using the SLE Disease Activity Index 2000 (SLEDAI-2K, range: 0–105), and damage was calculated using the SLICC damage index (SDI, range: 0–47). All information was later extracted from medical records. If information regarding SLEDAI-2K or SDI was missing, it was considered absent. Questionnaires were filled in by patients one day prior to the multidisciplinary assessment at the NPSLE clinic.

### Psychiatric diagnoses

Psychiatric diagnoses, which included both DSM-IV and DSM-5 diagnoses, were extracted from the medical records of the psychiatric part of the multidisciplinary assessment. All diagnoses were recoded according to DSM-5.^3^

### Cognitive dysfunction

All patients underwent a 1-hour standardized neuropsychological assessment (including the Minimal Mental State Exam, Wechsler Memory Scale, STROOP color and word test, and Trail Making Test). Cognitive dysfunction was considered present if the conclusion as reported in the medical record of the clinical neuropsychologist defined the presence of dysfunction in one or more cognitive domains.

### Dissociation

Dissociation was measured using the second (Dutch) version of the DES, a translated and validated questionnaire for screening the presence of dissociative disorders.^4^ It consists of 28 questions regarding dissociative experiences in daily life, which are rated on a scale from 0% (none of the time) to 100% (all of the time). The mean dissociation score is calculated by dividing the sum of percentages by 28 (range: 0–100). Scores ≥ 25 are suggestive of a dissociative disorder.^14^

Scores of the DES can be separated in different categories: amnesia, absorption/imagination, and depersonalization/derealization.^15^ In addition, question 28 specifically assesses the presence of a fog; this score was reported separately. A score of ≥ 30 on this question was considered indicative for the presence of a dissociative fog.

### Missing data

The DES was missing in 34 patients (9%), and information on neuropsychological status was missing in 11 patients (3%). Education level was missing in 3.6% and psychiatric assessment in 0.3%.

## Statistical analyses

Association between the presence of an inflammatory phenotype and dissociation was studied using multiple linear regression analysis corrected for age, sex, and education level. Because of non-normal distribution, the average DES score was natural log transformed. The result is presented as back-transformed B and 95% confidence interval (CI). Dissociation was compared between patients with/without prednisone using the Mann–Whitney test.

## Sensitivity analyses

First, patients with a dissociative disorder were excluded from the analysis. Second, patients with solely peripheral nervous system involvement were excluded. Lastly, multiple imputation using chained equations was performed (for details, see the Supplementary File).

All analyses were performed using STATA 16. College Station, TX: StataCorp LLC.

## Results

### Patient characteristics

Between 2007–2019, 577 patients visited the NPSLE clinic, of which 371 patients met the inclusion criteria (see Supplementary Figure 1)*.* Information on DES was available for 337 patients (91%), of which 69 patients (20%) had neuropsychiatric symptoms of inflammatory origin (inflammatory or combined NPSLE phenotype). Of the 268 patients with a non-inflammatory origin, 28 patients had ischemic NPSLE (10%) and 240 had minor/non-NPSLE (90%). The mean age in the total study population was 44 ± 14 years, and the majority was female (87%), as shown in Table 1*.* Baseline characteristics of patients with/without questionnaire were similar. All NPSLE syndromes are described in Supplementary Table 1.

**Table 1.** Baseline characteristics of patients with systemic lupus erythematosus and neuropsychiatric symptoms visiting the NPSLE clinic between 2007–2019.

	SLE patients with neuropsychiatric symptoms (*n* = 337)
*Female*	293 (87)
*Age* (years)	43.4 ± 13.5
*Ethnicity/Race* (% Caucasian)	242 (72)
*SLE duration* (years)	4.5 [0–40]
*ACR 1997 criteria*	
Malar rash	133 (40)
Discoid rash	58 (17)
Photosensitivity	170 (50)
Oral ulcers	144 (43)
Nonerosive arthritis	204 (61)
Pleuritis or pericarditis	86 (26)
Renal disorder	90 (27)
Neurologic disorder	42 (13)
Hematologic disorder	163 (48)
Immunologic disorder	261 (77)
Positive ANA	329 (98)
*Disease activity* (SLEDAI-2K)	
No/mild (0–5)	207 (61)
Moderate (6–11)	85 (25)
Severe (≥ 12)	45 (13)
*SDI (median, range)*	1 (0–11)
*Education level (years)*	
Low (0–6)	14 (4)
Middle (6–12)	207 (61)
High (> 12)	104 (31)
Unknown	12 (4)
*NPSLE*	
Inflammatory	47 (14)
Ischemic	28 (8)
Combined	22 (7)

Results are presented as *n* (%), mean ± SD or median [range]. NPSLE = neuropsychiatric systemic lupus erythematosus, SLEDAI-2K = Systemic Lupus Erythematosus Disease Activity Index-2000; SDI = SLICC Damage Index.

A psychiatric diagnosis according to DSM-5 classification was present in 141 patients (42%). The most diagnosed disorders were depressive disorder (22%), anxiety disorder (5%), and trauma- and stressor-related disorders (5%). Cognitive dysfunction was present in 41% of patients. 135 patients (40%) used psychotropic medication, most frequently antidepressants and benzodiazepines (both 18%). Prednisone was used by 182 patients (54%).

### Dissociation

Median dissociation on the DES was 7.1 (range: 0–75) in the total group. In patients with an inflammatory phenotype, median dissociation was 6.4 (range: 0–75) vs 7.5 (range: 0–66) in the non-inflammatory phenotype. No association was found between the presence of neuropsychiatric symptoms of inflammatory origin and dissociation (B: 0.94 (95% CI: 0.83; 1.07)). The use of prednisone also did not influence the level of dissociation: median dissociation score was 6.8 vs 7.5 in patients not taking prednisone (*p* = 0.98).

In total, 35 patients (10%) had a dissociation score ≥ 25: 5 patients with an inflammatory phenotype (7%) and 30 non-inflammatory patients (11%). A comparison of baseline characteristics between patients with and without a dissociation score ≥ 25 revealed that especially depression (49 vs 19%) and psychosis (17% vs 2%) were more common in patients with a high score on the DES (see Supplementary Table 2). Median and ranges of the different subscores of the DES are described in Table 2*.* The most common type of dissociation was absorption/imagination in all patients (median: 12, range: 0–75). The median score on dissociative fog was 0 (range: 0–100), and 43 patients (13%) had a positive score on this question, of which 5 patients with an inflammatory phenotype (8%) and 38 non-inflammatory patients (14%).

**Table 2.** Presence of dissociation in patients with SLE and neuropsychiatric symptoms of different origins.

	Total cohort (*n* = 337)	Inflammatory phenotype (*n* = 69)	Non-inflammatory phenotype (*n* = 268)
*Dissociative Experience Scale*			
Median score	7.1 (0–75)	6.4 (0–75)	7.5 (0–66)
*Domain scores*			
Amnesia	5 (0–76)	4 (0–76)	5 (0–68)
Absorption/imagination	12 (0–75)	9.1 (0–73)	12.7 (0–7)
Depersonalization/derealization	1.4 (0–84)	1.4 (0–84)	1.4 (0–73)
Dissociative fog^a^	0 (0–100)	0 (0–100)	0 (0–100)

DES = Dissociative Experience Scale; NPSLE = neuropsychiatric systemic lupus erythematosus.

^a^Dissociative fog = question 28 of the DES.

## Sensitivity analyses

Two patients in the non-inflammatory group had a dissociative disorder according to DSM-5. Excluding these patients yielded similar results as the main analysis. In addition, exclusion of patients with solely peripheral nervous system (*n* = 5) also did not alter the results (B: 0.95 (95% CI: 0.83; 1.09)). After multiple imputation, the main results did not alter (see Supplementary Tables 3 and 4).

## Discussion

In this study, we analyzed the prevalence of dissociative symptoms in patients with SLE and neuropsychiatric symptoms, and demonstrated that high levels of dissociative symptoms (DES score ≥ 25) were present in 10% and dissociative fog in 13% of patients.

In the general population, adults have scores on the DES ranging between 4.4–14.^16^ We demonstrate a similar level of dissociation in our study population, contrary to our hypothesis. As patients may present with psychiatric symptoms to our clinic and many psychiatric diagnoses have been associated with increased dissociation,^11^ we expected to find more dissociation in our patient population. Although psychiatric disorders were more common in patients with a DES score ≥ 25, in general, DES scores were low. Only one study has previously investigated dissociation in SLE patients.^17^ Based on an arbitrary lower cut-off score of ≥ 15 on the DES, 47.5% showed signs of dissociation. Using a similar cut-off score in our population, dissociation was less frequent: only in 25% of patients. This large difference is most likely explained by the self-referral and the small study population (*n* = 40) in the mentioned study.^17^ We are the first to study dissociation in a large SLE population and in SLE patients that specifically present with neuropsychiatric symptoms. It is thought that dissociation is associated with inflammation,^10^ but we show that dissociation has the same prevalence in patients with inflammatory and non-inflammatory neuropsychiatric symptoms. We could therefore not confirm this hypothesis regarding the relationship between the presence of inflammation and dissociation.

As dissociative symptoms are uncommon and dissociative fog is only reported in 13% of patients, we assume that dissociation is not an important component of lupus fog. The unclarity regarding the exact definition and prevalence of lupus fog remains, which leads to the question whether similar symptoms in other diseases might provide more insight. Brain fog has indeed been described in several neuroimmune diseases, celiac disease,^18^ and chronic fatigue syndrome.^19^ In these diseases, fog is thought to be associated with mental fatigue and/or (mild) cognitive impairment, but extensive investigations are lacking. As cognitive impairment is frequently diagnosed in patients with SLE, the existing assumptions regarding the relationship between cognitive dysfunction and lupus fog should be further investigated. However, based on previous observations, cognitive dysfunction will probably also not capture the entire entity of ‘lupus fog’. In rheumatological practice, fog is considered very specific for lupus. However, cognitive dysfunction was eliminated in the first round of the selection process of the new classification criteria for SLE, indicating that cognitive dysfunction was *not* sufficiently specific for lupus.^20^ An approach such as the Delphi method should be applied, in which both lupus patients and experts are involved in gaining consensus regarding the definition of lupus fog. By defining lupus fog more consistently, recognition and treatment of this symptom will be enabled.

The strength of this study is that it is the first to investigate dissociation in a well-defined population of SLE patients with neuropsychiatric symptoms. All patients underwent standardized neuropsychological and psychiatric evaluation, providing context for the interpretation of dissociative symptoms in this patient population.

There are also limitations. Most importantly, patients were not directly asked whether they suffered from a ‘lupus fog’, and therefore, a direct comparison of dissociation and lupus fog was impossible. However, we obtained a first insight into the presence of a specific type of fog (dissociative fog) in patients with lupus. Furthermore, patients included in this study were referred to our tertiary referral center for neuropsychiatric symptoms and are therefore not a reflection of the general lupus population. Despite this specific selection based on neuropsychiatric symptoms, dissociation was infrequent. Therefore, we would expect even less dissociation in the general SLE population and that our conclusion therefore holds. Future studies are needed to demonstrate whether our results are reproducible and to determine the characteristics of ‘fog’ in relation to (NP)SLE, cognitive dysfunction, and psychiatric comorbidity.

In conclusion, we demonstrate that patients with SLE and neuropsychiatric symptoms (both inflammatory and non-inflammatory) have dissociative symptoms within the normal range and that dissociative fog is uncommon.

## Supplemental Material

sj-pdf-1-lup-10.1177_09612033211050347 – Supplemental Material for Dissociation in SLE: A part of lupus fog? Click here for additional data file. Supplemental Material, sj-pdf-1-lup-10.1177_21925682211049167 for Dissociation in SLE: A part of lupus fog? by Rory C Monahan, Anne ME Blonk, Esther Baptist, Huub AM Middelkoop, Margreet Kloppenburg, Tom WJ Huizinga, Nic J van der Wee and Gerda M Steup-Beekman in Lupus
