# Supplementary material for: Dissociation in SLE: A part of lupus fog?
Source: Lupus. 2021 Oct 29;30(13):2151–6. doi: 10.1177/09612033211050347 (PMC8647480; doi:10.1177/09612033211050347)
Supplement: sj-pdf-1-lup-10.1177_09612033211050347 – Supplemental Material for Dissociation in SLE: A part of lupus fog? [file sj-pdf-1-lup-10.1177_09612033211050347.pdf]

Supplementary files

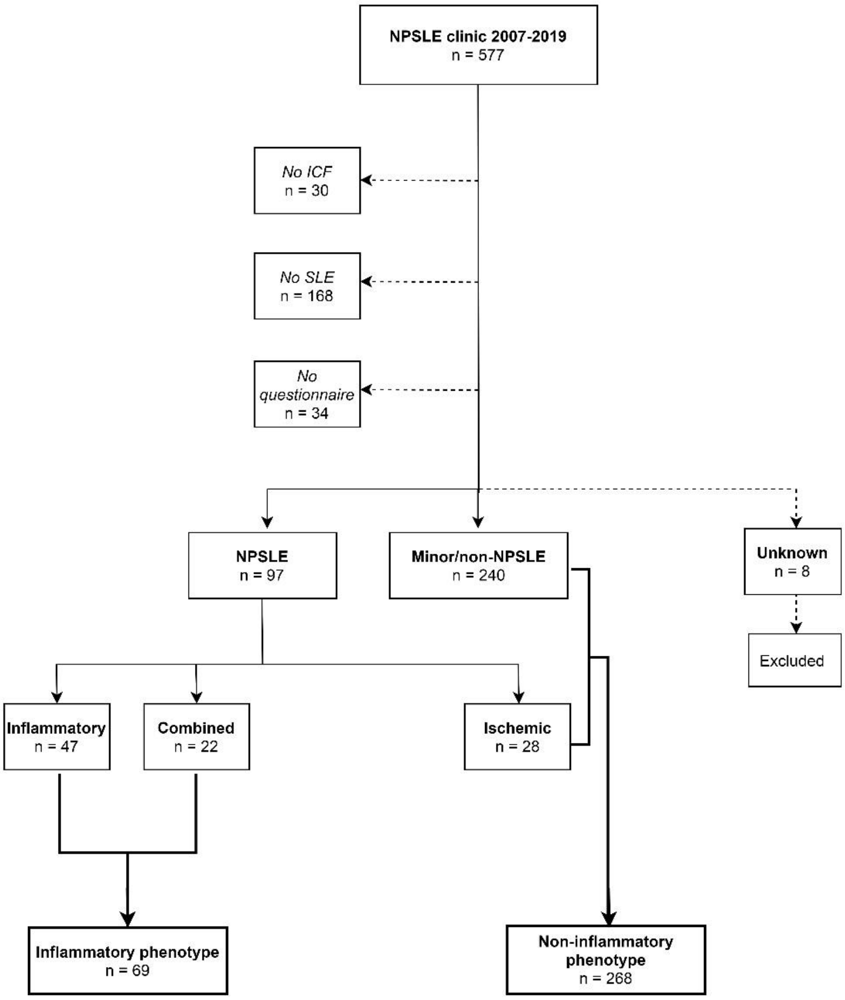

Supplementary Figure 1 Flow chart of patient inclusion

ICF = informed consent; *NPSLE = neuropsychiatric systemic lupus erythematosus, Unknown = unclear diagnosis (NPSLE yes/no)*

Supplementary Table 1 NPSLE syndromes according to 1999 ACR criteria of patients with SLE and neuropsychiatric symptoms attributed to SLE (NPSLE)

	**Inflammatory phenotype**[a] (n = 69)	**Ischemic phenotype** (n = 28)
**NPSLE syndrome**		
Aseptic meningitis	1 (1)	0 (0)
Cerebrovascular disease	19 (28)	26 (93)
Demyelinating syndrome	0 (0)	0 (0)
Headache	7 (10)	1 (3)
Movement disorder (chorea)	3 (4)	0 (0)
Myelopathy	7 (10)	0 (0)
Seizure disorders	5 (7)	3 (10)
Acute confusional state	6 (9)	0 (0)
Anxiety disorder	1 (1)	0 (0)
Cognitive dysfunction	32 (46)	2 (7)
Mood disorder	12 (17)	0 (0)
Psychosis	5 (7)	0 (0)
AIDP[b]	0 (0)	0 (0)
Autonomic disorder	0 (0)	0 (0)
Mononeuropathy	0 (0)	0 (0)
Myasthenia gravis	0 (0)	0 (0)
Neuropathy, cranial	6 (9)	0 (0)
Plexopathy	0 (0)	0 (0)
Polyneuropathy	5 (7)	0 (0)
Other[c]	18 (26)	0 (0)

Data is presented as n (%)

[a] Patients with NPSLE of inflammatory origin (n = 69): inflammatory or combined NPSLE phenotype.

[b] Acute inflammatory demyelinating polyneuropathy.

[c] Other NPSLE symptoms: cerebral vasculitis (n = 7), organic brain syndrome (n = 3), lethargy (n = 1) visual disturbance other than optic neuritis (n =1), apraxia (n =1), walking disorder (n =2), motor disorder left arm (n =1), paresis left arm and dysarthria (n = 1), increased intracranial pressure (n =1).

Supplementary Table 2 Comparison of baseline characteristics between patients with and without pathological dissociation scores

	**DES < 25**<br>n = 302	**DES ≥ 25**<br>n = 35	
**Female**	261 (82)	32 (91)	p = 0.41
**Age (years)**	44 ± 13	39 ± 14	p = 0.05
**SLE duration (years)**	5 [0 – 40]	4 [0 – 22]	p = 0.62
**Disease activity (SLEDAI-2K)**	4 [0 – 34]	4 [0 – 34]	p = 0.77
**Education level**			p = 0.51
Low	0	14	
Middle	182	23	
High	94	2	
Missing	2	10	
**NPSLE**	87 (29)	10 (29)	p = 0.977
**Cognitive dysfunction**	121 (40)	18 (51)	p = 0.20
**Depression**	57 (19)	17 (49)	p = 0.00
**Anxiety**	15 (5)	1 (3)	p = 0.58
**Psychosis**	7 (2)	6 (17)	p = 0.00
**Trauma- and stressor related disorders**	14 (5)	2 (6)	p = 0.78
**Dissociative disorders**	0 (0)	2 (6)	p = 0.00

Results are presented as n(%), mean ± sd, median [range]

Sensitivity analysis multiple imputation

A sensitivity analysis was performed with multiple imputation using chained equation in STATA version 16.0. Sex, age, ACR criteria, disease duration, disease activity score (SLEDAI-2K), damage index (SDI), NPSLE and NPSLE phenotype, DSM-5 diagnoses and medication use were used for imputation (n = 50) using predictive mean matching.

Supplementary Table 3 Presence of dissociation in patients with SLE and neuropsychiatric symptoms, sensitivity analysis

	**Complete case** (n = 337)	**After imputation** (n = 371)
**Dissociative Experience Scale**		
Median score	7.1	7.6 (95% CI: 6.6 – 8.6)
Domain scores		
Amnesia	5	5 (95% CI: 4.0 – 6.0)
Absorption/imagination	12	12.1 (95% CI: 10.2 -14.0)
Depersonalization/derealization	1.4	1.4 (95% CI: NA)

Supplementary Table 4 Comparison of dissociative symptoms in patients with SLE and neuropsychiatric symptoms of an inflammatory vs non-inflammatory phenotype

	**Complete case** (n = 337)	**After imputation** (n = 371)
**Dissociation, inflammatory vs non-inflammatory**	β: 0.93 (0.83; 1.07)	β: 0.92 (0.73; 1.17)
